# The influence of positivity and self-efficacy beliefs on family functioning among young adults in Italy and Colombia

**DOI:** 10.3389/fpsyg.2024.1411263

**Published:** 2024-09-18

**Authors:** Roberto Baiocco, Jessica Pistella, Maryluz Gomez Plata, Mara Morelli, Stefano Isolani, Maryoris Elena Zapata Zabala, Kattia Paola Cabas Hoyos, Liliana Maria Uribe Tirado, Marcela Sucel Ruiz Garcia, Carmelina Paba Barbosa, Antonio Zuffiano, Maria Gerbino, Fiorenzo Laghi, Concetta Pastorelli

**Affiliations:** ^1^Department of Developmental and Social Psychology, Faculty of Medicine and Psychology, Sapienza University of Rome, Rome, Italy; ^2^University of Magdalena, Santa Marta, Colombia; ^3^Department of Dynamic, Clinical Psychology and Health, Faculty of Medicine and Psychology, Sapienza University of Rome, Rome, Italy; ^4^University of San Buenaventura Medellin, Medellín, Colombia; ^5^Department of Psychology, Sapienza University of Rome, Rome, Italy

**Keywords:** positivity, self-efficacy beliefs, family functioning, sexual orientation, Colombia

## Abstract

**Background:**

Research suggests that positivity and self-efficacy beliefs may impact adaptive behavior and developmental outcomes, such as social adjustment and subjective wellbeing. The present study explored the effect of positive dimensions (positivity and self-efficacy beliefs) and individual characteristics (gender, type of country, age, and sexual orientation) on family cohesion and flexibility in a group of Colombian and Italian young adults.

**Method:**

An online survey was administered to 949 Colombian and 2,073 Italian people aged between 18 and 40 years (*M*_age_ = 24.3; SD_age_ = 4.5; 67% women). A mediational model was performed to test the influence of positivity on family functioning via the mediational role of self-efficacy beliefs, analyzing the moderated effects of gender, type of country, sexual orientation, and age.

**Results:**

Filial self-efficacy mediated the effect of positivity on family functioning, showing stronger paths in men and Colombian participants than in women and Italian counterparts. Regulatory self-efficacy mediated the associations between positivity and family functioning for both genders and types of countries.

**Conclusion:**

The results suggest that positivity and self-efficacy beliefs may allow families to engage in more adaptive family functioning across countries and genders. Further research should focus on implications from a cross-national perspective to examine other culture-specific factors that may impact family adjustment.

## Introduction

Positivity (POS) is defined as a stable self-evaluative disposition that captures people’s tendency to view their experience through a positive lens (Caprara et al., 2019) and a determinant of happiness (Caprara et al., 2017). Many studies explored the role of POS and self-efficacy beliefs—a personal judgment of one’s s capability to succeed in specific domains of functioning, such as the emotional domain (Thartori et al., 2021). However, to our knowledge, no studies examined the relations among POS, self-efficacy beliefs in filial, social, and regulatory domains, and family functioning (i.e., family cohesion and flexibility) in young adults. Thus, the present study aimed to assess the impact of POS on family functioning both directly and indirectly via the mediational role of self-efficacy beliefs (filial, social, and regulatory), examining the moderated effects of gender, type of country (Italy vs. Colombia), sexual orientation, and age.

POS represents the human tendency to construe subjective individual experiences from a positive outlook. Several authors recognized that the phenomenological expressions of POS are self-esteem, life satisfaction, and optimism (Caprara et al., 2019). The positive view of themselves, their life, and their future has a biological function, given that such a basic trait provides coping strategies to face life’s stressful and adverse events (Caprara et al., 2010). Diener et al. (2000) referred to POS as a propensity to evaluate aspects of life in general as good despite the environmental change by predisposing people to positive emotional experiences and enhancing positive feelings.

Indeed, a positive disposition helps to maintain an adequate level of vigilance toward threats and cope effectively with uncertainties and difficulties, providing the individual ability to adapt successfully to changing and stressful environmental contingencies. Many studies demonstrated that people with high levels of POS presented positive outcomes, such as good physical health, hedonic balance, social support (Caprara et al., 2010), positive affectivity (Alessandri et al., 2015), ego-resiliency (Milioni et al., 2016), friendship quality (Zhou et al., 2022), positive interpersonal relationships (Baumeister et al., 2003; Lyubomirsky et al., 2005; Scheier et al., 1994), and social adjustment (Caprara et al., 2019).

Previous research has pointed to the associations of POS with other adaptive individual characteristics, such as self-efficacy beliefs, which Bandura (2008) defined as a personal judgment of one’s capabilities to organize and execute the courses of action required to produce given attainments. High levels of POS in life could be related to higher self-efficacy and general wellbeing despite the adverse change (Thartori et al., 2021). For instance, regarding the association between POS and self-efficacy beliefs, studies examining people’s tendencies to overestimate their performance found that POS correlated with individuals’ tendency to perceive their academic performance better than the average, independent of their previous objective academic achievements (Caprara et al., 2012a, 2012b).

### Positivity, self-efficacy beliefs, and family functioning

Previous research explored the impact of POS and self-efficacy beliefs on different outcomes. A recent study (Thartori et al., 2021) found that POS contributes to better regulating participants’ emotions during the stressful situation of the COVID-19 pandemic, highlighting that self-efficacy beliefs may represent a mediator that significantly contributes to turning POS into specific behaviors, such as anxiety, depressive symptoms, and adequate coping strategies. According to the research that identified self-efficacy beliefs as a mediator in the relationship between POS and adaptive outcomes, to our knowledge, no studies examined the relationship between POS, self-efficacy beliefs, and family functioning and adjustment.

Family functioning refers to effective emotional bonding between family members, the use of family rules, family communication, and external management (Fang et al., 2004): It regards how family members interact and work together to achieve common goals and outcomes (Yuan et al., 2019). Specifically, previous research found that family cohesion (i.e., emotional connectedness) and family flexibility (i.e., change in leadership and role relationships) are linearly correlated with good family functioning and are central features of high family communication and satisfaction. Research shows that when family members’ communication is good, the family is closer, more loving, and more flexible in solving problems (Barnes and Olson, 1985; Olson, 2000).

A recent systematic review demonstrated that positive family functioning significantly correlates with subjective wellbeing over the course of development (Izzo et al., 2022). In particular, higher POS was related to better family adjustment (Diener and Chan, 2011). Given that POS builds personal and relational resources in family members as they respond to ongoing challenges and seek their resolution (Park, 2010), it is reasonable to assume that people high in POS might be inclined to actively (and enthusiastically) participate in family activities. POS can positively contribute to developing a sense of interconnectedness with family members, increasing their willingness to engage in cohesive and adaptive actions for the family’s wellbeing.

Thus, we reasoned that an individual with a positive approach to life is relatively likely to engage in positive relationships and actively promote positive social and relational environments such as the family system (Luengo Kanacri et al., 2017). Similarly, people predisposed to view themselves and their world in a positive way are more likely to perceive their family environment optimistically. The contribution of self-efficacy has been recognized across a variety of domains of human functioning, such as health, academic achievement, and social relationships (Caprara and Steca, 2006): Self-efficacy beliefs help to sustain motivation, positive emotional experiences, coping strategies, and regulate the behaviors per their personal goals.

Even if POS and self-efficacy beliefs are two dimensions concerned with the self-evaluative system (Caprara et al., 2019), they may operate at different levels to predict an individual’s and family’s functioning. Specifically, POS works at a basic level by predisposing people to face the challenges of the human condition, orienting their attention to resources, opportunities, and success, rather than weaknesses, difficulties, and adverse outcomes of the family system. At the same time, self-efficacy beliefs likely operate at an intermediate level by allowing people to transform their positive orientation into effective actions to create a more positive family climate.

Thus, POS (Diener and Chan, 2011) may contribute directly and indirectly, i.e., through self-efficacy beliefs (Caprara and Steca, 2006), to developing good family functioning and functional family relationships (e.g., high-income family coherence and low family conflict). Individuals inclined to evaluate themselves, their lives, and the future in a positive outlook may be more willing to display more self-efficacy and stable/positive family functioning. Indeed, POS is a basic trait that pervasively affects how individuals appraise, view, and construe their own experience and then act: We reasoned that individuals who have a self-evaluative tendency to view themselves, their own life, and the future under a positive outlook might better regulate their behaviors, by structuring positive and harmonious family relationships based on cohesion and flexibility among family members.

POS, self-efficacy beliefs, and family functioning could vary based on some individual variables. For instance, previous studies suggest that men (Thartori et al., 2021) and older young adults (Fagnani et al., 2014) reported higher levels of POS than women and younger adults. Similarly, men and older young adults reported higher levels of self-efficacy than women (Caprara and Steca, 2006) and younger adults (Caprara et al., 2003). During young adulthood, young women described their families as more cohesive and emotionally closer than young men (Allen and Stoltenberg, 1995; Valls-Vidal et al., 2016).

Sexual orientation could be another sensitive variable in evaluating the relationship between individual positive dimensions and family functioning. For instance, the literature identifies sexual minority people’s coming out to parents as a significant challenge that can revolutionize family relationships and may be associated with decreased psychological wellbeing and the undoing of family bonds over time (Baiocco et al., 2020; Gonzalez et al., 2013; Pistella et al., 2020). Again, previous research evidenced that sexual minority people presented lower self-efficacy beliefs than their heterosexual counterparts (Tovar-Murray et al., 2021).

### The present study

The current research is based on a literature review of the positive psychology contributions (Seligman and Csikszentmihalyi, 2000), focusing on personal and environmental determinants of individual wellbeing in promoting resources and positive aspects of themselves (Cabanas and Sánchez-González, 2016). According to previous research, POS (Caprara et al., 2019) and self-efficacy (Alessandri et al., 2015) represent the primary psychological resources for managing stressful events and adversities (Thartori et al., 2021), also playing a pivotal role in diverse positive outcomes, such as social adjustment (Caprara et al., 2019).

We aimed to assess the protective role of POS and filial, social, and regulatory self-efficacy in family adjustment in terms of family cohesion and flexibility, given that POS represents a disposition that prepares people to deal with life with a positive attitude from the beginning. Indeed, although both POS (Caprara et al., 2012a) and self-efficacy (Bandura, 2008) are two core aspects of the broader self-system, some scholars highlighted that high levels of POS might impact self-efficacy and family adjustment (Diener and Chan, 2011).

To our knowledge, no studies examined the relationship between POS, self-efficacy, and family functioning in different cultural contexts, such as Colombia and Italy. Cross-cultural research (Schlosser, 2006) found that Colombian people consider agreeableness (i.e., benevolent ways to approach life and others) and a human orientation (i.e., the degree to which members of a society are altruistic and kind to others) as valuable dimensions of life (Ardila et al., 2012). In addition, Colombian culture is characterized by collectivistic, rather than individualistic values, such as the Italian one, and by an optimistic approach toward life and the future (Griffith et al., 1998; Luengo Kanacri et al., 2017) compared to other Western countries.

However, individuals from collectivist cultures did not report fewer efficacy beliefs, goals, and personal accomplishments than individualistic countries (Basili et al., 2020). Regarding family functioning, Colombia and Italy are countries defined by conservative and family-oriented values (Baiocco and Pistella, 2019; Luengo Kanacri et al., 2017): Previous studies indicated that better family functioning might be a protective factor because it is associated with lower odds of poor mental health in Colombia (Tamayo-Aguledo et al., 2022) and Italy (Baiocco et al., 2020). For these reasons, the study aimed to improve our knowledge about POS, self-efficacy, and family functioning in Colombian and Italian young adults.

**Figure 1:**
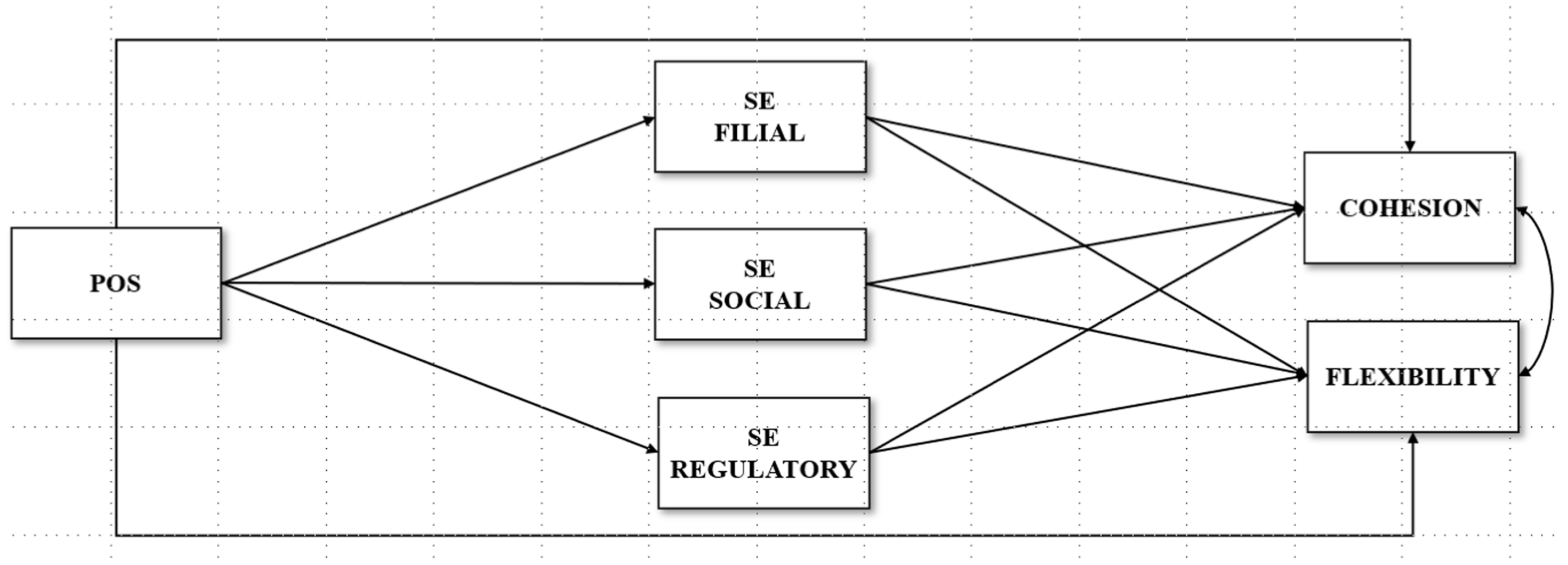
Hypothesized model. Gender, type of country, sexual orientation, and age are included as moderators in the model. POS, positivity; SE, self-efficacy.

In this study, we tested a mediational model in which POS exerts its influence on family cohesion and family flexibility both directly and indirectly via the mediational role of self-efficacy beliefs in the three domains (filial, social, and regulatory), examining the moderated effects of gender, type of country (Colombia vs. Italy), sexual orientation, and age. The conceptual model is depicted in Figure 1.

Specifically, we formulated the following hypotheses: POS positively predicts high levels of family cohesion and flexibility (Hypothesis 1a) and positively predicts one’s perceived self-efficacy beliefs (Hypothesis 1b); perceived self-efficacy beliefs (filial, social, and regulatory) positively predict high family cohesion and flexibility (Hypothesis 2); perceived self-efficacy beliefs (filial, social and regulatory) mediate the relations between POS and family cohesion and flexibility (Hypothesis 3).

Again, several studies highlighted how women (Allen and Stoltenberg, 1995; Valls-Vidal et al., 2016) and younger adults (Parra et al., 2015) reported higher levels of family cohesion and flexibility than men and older young adults. In turn, men and older young adults indicated higher levels of POS and self-efficacy beliefs than their counterparts. Thus, we explored whether gender and age could moderate the effects of POS and self-efficacy on family functioning. Finally, based on the literature that highlighted cultural differences between Colombia and Italy (Luengo Kanacri et al., 2017; Tamayo-Aguledo et al., 2022) and different levels of self-efficacy (Tovar-Murray et al., 2021), positive emotions, and family adjustment (Gonzalez et al., 2013) in sexual minority people compared to heterosexual young adults, we expected that the type of country and sexual orientation could be moderators in the relationship between POS, self-efficacy beliefs, and family functioning (Hypothesis 4).

## Methods

### Participants

Data collection in the present study was conducted between December 2019 and June 2021, in both Italy and Colombia, resulting in a total of 3,022 participants (2,073 from Italy and 949 from Colombia) aged between 18 and 40 years old (*M*_age_ = 24.32; SD_age_ = 4.46; 67.3% women). Overall, 87.3% of participants (*n* = 2,639) reported being exclusively heterosexual, whereas the remaining 12.7% (*n* = 383) reported being LGB+ (i.e., lesbian, gay, bisexual, and other non-heterosexual sexual orientation).

### Procedure

An English version of the questionnaire was shared between Italian and Colombian researchers: Each country worked on a language adaptation of the survey before starting data collection. The two final versions (Colombian and Italian) were compared, resulting in no specific differences between them. All recruited participants filled in an online survey hosted by the Unipark platform through a snowball sampling procedure: They were asked to share the survey link among their contacts and social networks. Informed consent was obtained on the first page of the survey, where information about the project was offered.

Participation was voluntary and anonymous, and only the questionnaires filled out were considered valid among all the participants reached. The response rate was 95%. The duration of the survey was 20–25 min. The study was conducted according to the guidelines of the Declaration of Helsinki and approved by the Ethics Committee of the author’s institution. Data are available upon request in compliance with the General Data Protection Regulation (GDPR; Regulation EU2016/679).

### Instruments

**Socio-demographic variables**: Respondents provided information about their age, gender (men = 0; women = 1), type of country (Colombia = 0; Italy = 1), sexual orientation (heterosexual = 1; bisexual = 2; gay man = 3; lesbian woman = 4; other non-heterosexual sexual identities such as pansexual, asexual, or demisexual = 5). According to previous research on this topic (Pistella et al., 2020), participants were categorized as exclusively heterosexual (who answered 1) and LGB+ people (who answered from 2 to 5).

**Positivity** (POS; Caprara et al., 2012a): The positivity scale was used to assess participants’ tendency to perceive themselves, their lives, and their future with a positive outlook. The scale comprises eight items rated using a 5-point Likert scale ranging from 1 (strongly disagree) to 5 (strongly agree). An example item is “I have great faith in the future.” Higher scores reflect greater positivity. The scale has a unidimensional structure across several cultural contexts (Heikamp et al., 2014). The alpha reliability coefficient was.87.

**Self-efficacy Beliefs** (Bandura et al., 2003): Participants rated 19 items measuring their perceived capabilities in different domains of functioning. In detail, ten items assessed participants’ perceived *filial self-efficacy* in terms of their ability to hold an open dialog with their parents and to influence their parents’ attitudes and behavior in a positive way. An example item is “I can talk with my parents about my feelings toward them.” Twelve *social self-efficacy* items measured participants’ ability to form and maintain social relationships, work cooperatively with others, voice their opinions, and manage different interpersonal conflicts. An example item is: “I can express my opinions when other peers disagree with me.” The remaining seven items evaluated *regulatory self-efficacy* to resist peer pressure to engage in high-risk activities involving alcohol, drugs, and transgressive behaviors. An example item is: “How well can you resist peer pressure to drink beer, wine, or liquor?” For each item, participants rated the strength of their beliefs on a 5-point response scale ranging from 1 (perceived inability) to 5 (complete self-assurance in one’s ability). Higher scores reflect greater self-efficacy beliefs. Alpha reliabilities were.89 (filial), 0.89 (social), and.86 (regulatory).

**Family Functioning** (Olson, 2000): Participants completed the short version of the Family Adaptability and Cohesion Evaluation Scale (FACES IV; Baiocco et al., 2013). The scale contains eight items assessing Olson’s model’s two balanced scales (cohesion and flexibility) on a 5-point Likert scale (1 = completely disagree; 5 = completely agree). *Cohesion* (four items) reflects family members’ connectedness or emotional bonding. An example item is “Family members are supportive of each other during difficult times.” *Flexibility* (four items) is the ability to change, be flexible, and adapt. An example item is “My family is able to adjust to change when necessary.” Higher scores reflect greater cohesion and flexibility. Alpha reliabilities were.85 (cohesion) and.73 (flexibility).

### Data analysis

To test the hypothesized mediational model, we used a path analytic framework in which POS was the primary independent variable exerting its influence on family cohesion and family flexibility both directly and indirectly via the mediational role of self-efficacy beliefs in the three domains (filial, social, and regulatory). Moreover, we also explored whether the primary effect of POS on the mediators and the outcome was moderated by gender, country, age, and sexual orientation. Mediated effects (*ab*) were tested via 95% confidence intervals computed using 5,000 bootstraps (Hayes and Scharkow, 2013). Parameters were estimated with maximum likelihood with robust standard errors (MLR) in M*Plus* 8.4 (Muthén and Muthén, 2017).

## Results

Regarding the total sample (*n* = 3,022), the general level of education was average, with 39.4% of Colombian people and 51.8% of Italian participants having at least a university degree. In comparison, 60.6% of Colombian participants and 48.2% of Italian people had completed primary or secondary school. Demographic characteristics disaggregated by type of country are reported in Table 1.

**Table 1:** Descriptive (means, standard deviations, and percentages) of the characteristics of the sample by country.

	Colombia (*n* = 949)	Italy (*n* = 2073)	Total sample (*n* = 3,022)	*F/χ* ^2^	*p*
	*M* (SD) or *n*(%)	*M* (SD) or *n*(%)	*M* (SD) or *n*(%)		
Gender (men)	356 (38%)	633 (31%)	989 (33%)	14.40	<0.001
Sexual orientation (LGB+ people)	166 (18%)	217 (11%)	383 (13%)	29.02	<0.001
Age	24.26 (5.69)	24.35 (3.77)	24.32 (4.46)	0.26	0.61

The F/χ^2^ refers to the country differences. Standard deviations and percentages are in parentheses. Gender (men = 0, women = 1); Country (Colombia = 0, Italy = 1); Sexual orientation = (heterosexual = 0, LGB+ = 1).

### Zero-order correlations

As reported in Table 2, POS was positively correlated with each type of self-efficacy belief, family cohesion, and family flexibility. Again, self-efficacy beliefs were positively and strongly associated with family cohesion and flexibility. Being women and Italian people were related to higher family cohesion and flexibility than their counterparts. Age and sexual orientation were slightly associated with POS, self-efficacy beliefs, and family outcomes.

**Table 2:** Zero-order correlations.

	(1)	(2)	(3)	(4)	(5)	(6)	(7)	(8)	(9)	(10)
(1) Gender	1									
(2) Country	0.070^*^	1								
(3) Sexual Orientation	−0.02	−0.10^*^	1							
(4) Age	−0.08^*^	0.01	−0.09^*^	1						
(5) Positivity	−0.01	−0.16^*^	−0.10^*^	0.14^*^	1					
(6) SE Filial	0.03	−0.20^*^	−0.09^*^	0.12^*^	0.42^*^	1				
(7) SE Social	−0.10^*^	−0.11^*^	−0.06^*^	0.06^*^	0.45^*^	0.46^*^	1			
(8) SE Regulatory	0.11^*^	−0.09^*^	−0.07^*^	0.07^*^	0.29^*^	0.34^*^	0.45^*^	1		
(9) Cohesion	0.13^*^	0.14^*^	−0.08^*^	0.06^*^	0.27^*^	0.43^*^	0.20^*^	0.20^*^	1	
(10) Flexibility	0.11^*^	0.12^*^	−0.07^*^	0.07^*^	0.25^*^	0.40^*^	0.20^*^	0.20^*^	0.63^*^	1

*p < 0.01; gender (men = 0, women = 1); country (Colombia = 0, Italy = 1); sexual orientation = (heterosexual = 0, LGB+ = 1).

### Path analysis

The mediational model depicted revealed that two significant interaction terms, “POS × country” and “POS × gender,” were consistently and significantly associated with both mediators and outcomes. As the other interaction terms (i.e., “POS × age” and “POS × sexual orientation”) were not statistically significant, they were removed from the analysis to ease the interpretation of the findings. Simple slope analyses indicated a consistent pattern of effects from POS to the three types of self-efficacy beliefs, in which the positive paths were stronger in Colombia than in Italy (see Table 3).

**Table 3:** Simple slope analysis for the interactions “POS × Country” and “POS × Gender.”

	Country		Gender	
	Colombia	Italy	Men	Women
POS → Cohesion	0.013	0.143*	0.043	0.113*
POS → Flexibility	0.071	0.071	0.029	0.112*
POS → SE Filial	0.441*	0.345*	0.429*	0.357*
POS → SE Social	0.429*	0.347*	0.427*	0.349*
POS → SE Regulatory	0.331*	0.241*	0.349*	0.224*

The asterisk (*) indicates that the simple slope was statistically significant (*p* < 0.05). All the effects reported in the table are unstandardized regression coefficients. POS, positivity; SE, self-efficacy.

Interestingly, we also found that the effect of POS on family cohesion was moderated by country, with POS showing a stronger effect in Italy than in Colombia. Concerning the interaction with gender, the simple slope analysis indicated a similar consistent pattern in which the positive effects of POS on the three types of self-efficacy beliefs were stronger in men than women. However, the positive effects of POS on family cohesion and family flexibility were slightly stronger in women than men (see Table 3). Thus, the results highlighted the direct effects of POS on family cohesion and flexibility, with stronger effects in women and Italian participants compared to men and Colombian counterparts.

Family cohesion and flexibility, in turn, were positively related to higher filial and regulatory self-efficacy levels. Interestingly, while controlling for the other types of self-efficacy, social self-efficacy did not show a significant effect on both outcomes. The full set of results of the mediational model is reported in Table 4.

**Table 4:** Standardized paths from the mediational model.

	Mediators			Final outcomes	
	SE filial	SE social	SE regulatory	Cohesion	Flexibility
Gender	0.047*	−0.097*	0.122*	0.091*	0.079*
Country	−0.143*	−0.028	−0.052*	0.231*	0.216*
Sexual orientation	−0.056*	−0.024	−0.043*	−0.002	0.000
Age	0.062*	−0.020	0.036	−0.001	0.003
POS	0.500	0.567*	0.418*	−0.027	0.040
POS x Gender	−0.062*	−0.077*	−0.108*	0.069*	0.096*
POS x Country	−0.080*	−0.079*	−0.075*	0.122*	—
SE Filial	—	—	—	0.425*	0.382*
SE Social	—	—	—	−0.036	−0.020
SE Regulatory	—	—	—	0.050*	0.052*

Gender (men = 0, women = 1); country (Colombia = 0, Italy = 1); sexual orientation = (heterosexual = 0, LGB+ = 1); POS, positivity; SE, self-efficacy. The asterisk (*) indicates that the standardized effect was statistically significant (*p* < 0.05). The Mplus output of this model is reported in the OSF at the following link: (blinded for peer review).

Considering gender differences, the analyses of the mediational effects showed that filial self-efficacy mediated the effect of POS on family flexibility and cohesion, yet these effects were slightly stronger for men (*ab* = 0.122, 95% CI: 0.102, 0.145, and *ab* = 0.160, 95% CI: 0.134, 0.189, respectively) than women (*ab* = 0.102, 95% CI: 0.086, 0.118, and *ab* = 0.133, 95% CI: 0.113, 0.154, respectively), and in Colombia (*ab* = 0.126, 95% CI: 0.104, 0.149, and *ab* = 0.165, 95% CI: 0.136, 0.195, respectively) compared to Italy (*ab* = 0.098, 95% CI: 0.084, 0.114, and *ab* = 0.129, 95% CI: 0.110, 0.148, respectively).

Regulatory self-efficacy also mediated the effects of POS on family flexibility and cohesion for both men (*ab* = 0.014, 95% CI: 0.005, 0.024; *ab* = 0.016, 95% CI: 0.005, 0.028, respectively) and women (*ab* = 0.012, 95% CI: 0.004, 0.021; *ab* = 0.012, 95% CI: 0.003, 0.021, respectively), although its mediational role was smaller than the role of filial self-efficacy. Similar mediated effects for regulatory self-efficacy were also obtained in Italy (*ab* = 0.009, 95% CI: 0.003, 0.016, and *ab* = 0.011, 95% CI: 0.003, 0.019, respectively) and Colombia (*ab* = 0.013, 95% CI: 0.004, 0.023, and *ab* = 0.015, 95% CI: 0.004, 0.027, respectively).

## Discussion

This study contributes to increasing the scientific knowledge related to POS, self-efficacy beliefs, and family functioning in young Italian and Colombian adults. Empirical data are essential in understanding the individual protective factors that may impact family adjustment in two family-oriented counties (Baiocco and Pistella, 2019; Luengo Kanacri et al., 2017), where the family is perceived as a critical institution in the provision of intergenerational care relations and closeness. Specifically, given that previous empirical findings support the view that POS plays a role in diverse positive outcomes, such as social adjustment (Caprara et al., 2019), an innovative aspect of the present study was investigating the protective role of POS and filial, social, and regulatory self-efficacy in family adjustment, in terms of family cohesion and flexibility.

The moderated mediational model revealed significant two-way interactions between POS and gender and between POS and country, considering all mediators and outcomes. First, the results highlighted the direct effects of POS on family cohesion and flexibility (Hypothesis 1a), with stronger effects in women and Italian participants than in men and Colombian counterparts. Positive associations emerged between POS and filial, social, and regulatory self-efficacy beliefs (Hypothesis 1b), and the positive paths were greater in men than women and in Colombia than Italy. The gender as moderator is in line with previous studies: Men presented higher levels of POS (Thartori et al., 2021) and self-efficacy beliefs (Caprara and Steca, 2006) than their counterparts, whereas others highlighted the relevance of POS in Colombian cultures (Griffith et al., 1998; Luengo Kanacri et al., 2017; Schlosser, 2006).

Results showed that higher filial and regulatory self-efficacy levels were positively associated with family cohesion and flexibility (Hypothesis 2). Social self-efficacy did not produce a significant effect when inserting the other types of self-efficacy beliefs. Thus, the final model suggested that filial self-efficacy mediated the effect of POS on family cohesion and flexibility (Hypothesis 3), showing that the relationships are stronger for men and Colombian participants than for women and Italian respondents.

Filial self-efficacy regards the perceived capability to communicate with their parents, express their inner problems, manage negative emotions during contrasts with their parents, and act assertively in such a way that their parents can develop positive attitudes and behaviors toward them. Therefore, the participants’ capability to maintain a good relationship with their parents by inducing parents to have positive opinions about them mediates the relationship between their POS and family cohesion and flexibility. Most research demonstrated that poorer perceived quality of the relationship with the parents predicted high levels of psychopathological difficulties (Izzo et al., 2022; Pinquart, 2017). At the same time, the present study highlighted the importance of filial self-efficacy in impacting young adults’ positive individual factors on family adjustment.

The mediation effect of filial self-efficacy is stronger in men than women: Studies showed that women reported greater levels of family cohesion and flexibility than men (Allen and Stoltenberg, 1995; Valls-Vidal et al., 2016), whereas men are higher in POS (Thartori et al., 2021) and general self-efficacy beliefs (Caprara and Steca, 2006). Thus, in men who participated in our research, their POS predicted family adjustment through the mediated role of the ability to maintain a positive relationship with their parents (filial self-efficacy). Women perceived their family system as more cohesive and flexible regardless of filial self-efficacy.

Regarding the moderated effect of the type of country, an explanation is that the Colombian people, regardless of gender, had higher levels of POS than other individualistic countries (Schlosser, 2006). For instance, previous studies consider Colombian people to have more benevolent and humane ways of approaching life and other people, and they are highly altruistic and kind toward others (Griffith et al., 1998; Luengo Kanacri et al., 2017). Filial self-efficacy in Colombian people could predict family adjustment due to the desire to help one’s system be more functioning, adaptive, cohesive, and flexible. These considerations about the Colombian cultures could partially explain the stronger association between the three types of self-efficacy beliefs and POS for Colombian participants than the Italian one. However, these explanations are only speculative because we did not consider prosocial or altruistic behavior in our research.

Moreover, the path analysis indicated that regulatory self-efficacy mediates the effect of POS on cohesion and flexibility (Hypothesis 3) among both men and women and Italian and Colombian participants, showing common paths between them. Regulatory self-efficacy consists of people’s perceived capability to self-regulate their behavior in transgressive contexts: Children’s capacity to resist peer pressure for engaging in transgressive conduct may play a relevant role when facing interactions with their parents, siblings, or other family members. High POS may facilitate the ability to resist peer pressure to engage in high-risk activities, and young adults may share their non-at-risk behaviors with their parents, reinforcing family cohesion and flexibility. Regarding gender and country differences, our results align with a recent study (Basili et al., 2020) that assessed the measurement invariance of the self-efficacy scale across two samples of Italian and Colombian adolescents, showing that boys and girls, and Italian and Colombian participants, reported similar levels of regulatory self-efficacy beliefs. Thus, assuming that such a mediational effect emerges for both genders and countries is plausible.

In contrast, the mediating role of social self-efficacy in the relationship between POS and family functioning, as well as the moderating role of age and sexual orientation, were not significant. A possible explanation is that social self-efficacy mainly regards building and maintaining social relationships, especially working cooperatively with peer groups or managing interpersonal peer conflicts. Given that the model tested the impact of some individual variables on family outcomes, this dimension appears less relevant than filial and regulatory self-efficacy beliefs.

The moderated roles of gender and type of country confirmed our hypothesis partially because age and sexual orientation were not statistically significant (Hypothesis 4). The lack of such significance is unsurprising because correlation analyses showed that age and sexual orientation were slightly associated with key variables. Although some studies evidenced a difference between emerging adulthood and older young adults in positive dimensions and family outcomes, other research highlighted that during young adulthood, such levels might remain stable over time, decreasing in different developmental ages (Parra et al., 2015).

Finally, even if studies reported that sexual minority people presented lower levels of POS (Gonzalez et al., 2013) and self-efficacy (Tovar-Murray et al., 2021) than their heterosexual counterparts, positive experiences (such as positive parents’ reactions to coming out and low discrimination levels) might be providing help to improve their positive and adaptive individual characteristics, such as resilience, self-esteem, and positive orientation toward their life in general (Pistella et al., 2019). However, we did not consider these variables in our research, so future studies should control for these confounding factors.

In conclusion, our results seem to suggest that high POS and self-efficacy levels may have allowed families to engage in more positive family interactions across countries and genders throughout development. Positive skills and emotions toward themselves, their life, and their future among young adults could account for the increased family adjustment in terms of high cohesion and flexibility levels. An ecological systems approach suggests that families must be equipped with the positive skills, resources, and support to develop cohesion, adaptability, and positive family-member relationships. Indeed, our results confirm that POS and self-efficacy beliefs may work at different levels in predicting family functioning. As suggested previously, POS may predispose individuals to face challenges, orienting their attention to resources and opportunities, rather than weak aspects of their family system. The tendency to perceive their family system as cohesive and flexible may reflect a disposition to interpret family experiences from a positive outlook. Filial and regulatory self-efficacy beliefs may allow people to perform practical actions to create a more positive family climate, contributing directly and indirectly to developing positive family functioning.

### Limitations and future directions

Despite several strengths, our study had several limitations. Analyses were cross-sectional, and only self-report instruments were used without a measure of social desirability. The sampling method may have reduced the generalizability of the findings. Specifically, snowball sampling may lead to a significant bias in population characteristics, even if our sample characteristics (e.g., gender, sexual orientation, level of education) are comparable to those reported in previous studies using young Italian and Colombian adults (Morelli et al., 2023). Indeed, the research was conducted in Colombia and Italy, and the results may not be appropriate for people living in other cultural contexts: Future research should better apply longitudinal designs to recognize correlates of family functioning over time. There may also have been a bias in the results about POS because people tend to perceive their family system as cohesive and flexible, reflecting family experiences from a positive perspective. Again, the study used a dichotomous measure of gender (men/women), and future studies should include contemporary gender identities such as transgender and non-binary ones. Future studies should consider relevant outcomes related to positive dimensions and family functioning, such as family composition, socioeconomic status, educational level, academic achievements, coming-out and parents’ reactions, at-risk behaviors, and altruistic and prosocial behaviors. Further investigation could examine the role of these variables in promoting POS, self-efficacy beliefs, and family functioning, differentiating for a variety of countries.

We hypothesized that individual POS levels influence family functioning and that personal self-efficacy beliefs may mediate this relationship. In the present data, the consistent directional pattern of associations we found supports the priority of POS for positive dimensions in the samples. However, our model does not permit strong inferences about causal effects. Our results do not exclude the possibility that family functioning and self-efficacy beliefs might contribute to POS, sometimes or under certain circumstances. Indeed, it is also possible that POS is affected by family (mal)adjustment; i.e., youth who feel high levels of family cohesion and flexibility tend to develop a more positive view of the world. Recent studies, for instance, have shown that bidirectional relations might operate between positivity and self-efficacy beliefs (Caprara et al., 2024). Thus, future studies should examine bidirectional relations among POS, self-efficacy beliefs, and family functioning. Finally, we also recognize that the moderation effects tested were largely exploratory, and future confirmatory studies are needed to ascertain their robustness before drawing definitive conclusions.

The research has important implications for the adjustment of young adults. Given the importance of considering positive dimensions for promoting adjustment and wellbeing, we underlined the relevance of POS and self-efficacy on family functioning across gender, cultural contexts, sexual orientation, and age. We believe the current research findings will have practical significance for clinicians, mental health professionals, educators, and researchers in developing their understanding of POS and self-efficacy beliefs and their influence on adaptive family functioning. It could be helpful to create an understanding of methods and practices for promoting filial and self-regulatory self-efficacy, which played a relevant role in the relationship between POS and adequate family functioning. POS and self-efficacy dimensions protect young people from risky behaviors, such as substance use, favoring positive family interactions and social adjustment (Bandura et al., 2003; Basili et al., 2020). It is essential to explore implications from a cross-national perspective to examine potential differences in POS, self-efficacy beliefs, and other culture-specific factors that may influence family functioning.

The exploratory nature of the study discourages assumptions about the direction of the path between POS, self-efficacy beliefs, and family functioning. Similarly, they operate together, given that a positive orientation probably prepares people to deal with life in a positive mood. Educational interventions should be developed to transfer the culture’s core values concerning POS, self-efficacy, and altruistic behaviors. Therefore, further attention is needed to study how cultural contexts (i.e., Colombia and Italy) moderate relations among POS, self-efficacy, and family functioning. Educational and intervention programs are also needed to detect potential behavioral risk factors (Caprara et al., 2014). Indeed, previous research suggested that people with high POS, self-efficacy beliefs, and adequate family functioning are less likely to engage in at-risk behaviors and activities.

## Data Availability

The raw data supporting the conclusions of this article will be made available by the authors, without undue reservation.
