# Supplementary material for: The influence of positivity and self-efficacy beliefs on family functioning among young adults in Italy and Colombia
Source: Front Psychol. 2024 Sep 18;15:1411263. doi: 10.3389/fpsyg.2024.1411263 (PMC11444974; doi:10.3389/fpsyg.2024.1411263)
Supplement: Supplementary file 1 [file Presentation_1.pdf]

# Appendix

## Positivity (POS; Caprara et al., 2012a)

1. I have great faith in the future.
2. Others are generally here for me when I need them.
3. I am satisfied with my life.
4. At times, the future seems unclear to me. (r)
5. I generally feel confident in myself.
6. I look forward to the future with hope and enthusiasm.
7. I feel I have many things to be proud of.
8. On the whole, I am satisfied with myself.

## Self-efficacy Beliefs (Bandura et al., 2003)

*Filial self-efficacy*
How well can you…
1. Express your gratitude to your parents for their efforts on your behalf
2. Talk with your parent about your personal problems
3. Prevent differences of opinion with your parents from turning into arguments
4. Talk with your parent about your feelings toward them
5. Get your parents to understand your point of view on matters when it differs from theirs
6. Accept your parent's criticism of you without feeling offended
7. Get your parents to pay attention to your needs even when they are preoccupied with their own problems
8. Involve your parents in important decisions about your future
9. Take into account your parents' suggestions when they differ from your preferences
10. Get your parents to trust your judgment and responsibilities

*Social self-efficacy*
How well can you…
1. Participate in discussions that occur in groups.
2. Learn new sports.
3. Do physical exercise.
4. Learn what it takes to be part of a sports team (e.g., basketball, soccer, volleyball, etc.).
5. Realize what you expect of yourself.
6. Make friends with girls.
7. Make friends with boys.
8. Talk and share your opinion if you are with your friends to discuss something.
9. Work in a group.
10. Express your opinions when other peers disagree with you.
11. Stand up for your rights when you are mistreated.
12. Stand up for yourself if someone annoys or teases you.

*Regulatory self-efficacy*

How well can you…

1. Resist peer pressure to do things that get you into trouble.
2. Resist the temptation to not go to school/university/work when you feel bored or annoyed.
3. Resist if your friends push you to smoke.
4. Resist getting hold of things that appeal to you but don't belong to you.
5. Resist peer pressure to drink beer, wine, or liquor.
6. Resist your friends' insistence that you do something forbidden.
7. Resist pressure from your friends to take drugs.

**Family Functioning (Olson, 2000)**

*Cohesion*

1. Family members feel very close to each other.
2. Family members are supportive of each other during difficult times.
3. Family members like to spend some of their free time with each other.
4. Although family members have individual interests, they still participate in family activities.

*Flexibility*

1. Our family tries new ways of dealing with problems.
2. Parents equally share leadership in our family.
3. We shift household responsibilities from person to person.
4. When problems arise, we compromise.
